# The Synthetic α-Bromo-2′,3,4,4′-Tetramethoxychalcone (α-Br-TMC) Inhibits the JAK/STAT Signaling Pathway

**DOI:** 10.1371/journal.pone.0090275

**Published:** 2014-03-03

**Authors:** Sophia Pinz, Samy Unser, Susanne Brueggemann, Elisabeth Besl, Nafisah Al-Rifai, Hermina Petkes, Sabine Amslinger, Anne Rascle

**Affiliations:** 1 Stat5 Signaling Research Group, Institute of Immunology, University of Regensburg, Regensburg, Germany; 2 Institute of Organic Chemistry, University of Regensburg, Regensburg, Germany; University of Regensburg, Germany

## Abstract

Signal transducer and activator of transcription STAT5 and its upstream activating kinase JAK2 are essential mediators of cytokine signaling. Their activity is normally tightly regulated and transient. However, constitutive activation of STAT5 is found in numerous cancers and a driving force for malignant transformation. We describe here the identification of the synthetic chalcone α-Br-2′,3,4,4′-tetramethoxychalcone (α-Br-TMC) as a novel JAK/STAT inhibitor. Using the non-transformed IL-3-dependent B cell line Ba/F3 and its oncogenic derivative Ba/F3-1*6 expressing constitutively activated STAT5, we show that α-Br-TMC targets the JAK/STAT pathway at multiple levels, inhibiting both JAK2 and STAT5 phosphorylation. Moreover, α-Br-TMC alters the mobility of STAT5A/B proteins in SDS-PAGE, indicating a change in their post-translational modification state. These alterations correlate with a decreased association of STAT5 and RNA polymerase II with STAT5 target genes in chromatin immunoprecipitation assays. Interestingly, expression of STAT5 target genes such as *Cis* and *c-Myc* was differentially regulated by α-Br-TMC in normal and cancer cells. While both genes were inhibited in IL-3-stimulated Ba/F3 cells, expression of the oncogene *c-Myc* was down-regulated and that of the tumor suppressor gene *Cis* was up-regulated in transformed Ba/F3-1*6 cells. The synthetic chalcone α-Br-TMC might therefore represent a promising novel anticancer agent for therapeutic intervention in STAT5-associated malignancies.

## Introduction

The signal transducer and activator of transcription STAT5 is a key regulator of immune responses, cell proliferation, differentiation, survival and oncogenesis [1], [2]. STAT5 proteins normally reside as latent transcription factors in the cytoplasm of unstimulated cells. Following cytokine, growth factor or hormone stimulation, the receptor-associated JAK2 tyrosine kinase gets auto-phosphorylated (trans-phosphorylation) before it phosphorylates the receptor intracellular domain, creating docking sites for STAT5, which is in turn phosphorylated by activated JAK2. STAT5 phosphorylation allows its dimerization and translocation into the nucleus where it binds to specific recognition sites, and ultimately activates transcription of specific target genes (e.g. *c-Myc*, *Pim-1*, *Bcl-x*, *Cis*) [1], [3]–[5]. STAT5 activity is regulated through multiple post-translational modifications [1], [6]–[8] and through interactions with cofactors, other transcription factors or STAT5 itself (tetramerization) [1], [9]–[15]. STAT5 activation under physiological conditions is rapid and transient. Attenuation of the STAT5 response is controlled mainly through dephosphorylation by specific phosphatases (SHP-1) and a negative feedback loop mediated by proteins of the SOCS family (CIS, SOCS-1/-3) [16], [17]. However, constitutively activated STAT5 (caSTAT5) has been observed in a wide variety of human cancers, from hematologic malignancies (leukemia, lymphoma, myeloma) to solid tumors such as breast cancer, squamous cell carcinoma of the head and neck (SCCHN) or melanoma [16]–[18]. Constitutive activation of STAT5 directly contributes to oncogenesis, mainly through the up-regulation of oncogenes driving cell proliferation and survival (Bcl-x, c-Myc, Pim-1) [4], [19]–[23], but also through the down-regulation of tumor suppressor genes and inhibitors of the pathway, such as SHP-1 and CIS/SOCS family members, via epigenetic silencing [17], [18], [24]–[28].

Therefore, the JAK/STAT pathway in general and STAT5 proteins in particular represent attractive targets for therapeutic intervention in human cancers [18], [29]–[31]. Numerous inhibitors of the Janus Kinase family members (JAK1, JAK2, JAK3, Tyk2, and the JAK2V617F active mutant responsible for myeloproliferative neoplasia) and of STAT5 have been described [30], [32]–[45]. Most of the JAK inhibitors are small molecules acting as tyrosine kinase inhibitors by targeting their ATP-binding catalytic domain. The JAK1/JAK2 inhibitor Ruxolitinib has been approved by the U.S. Food and Drug Administration for the treatment of myelofibrosis while several others are being currently evaluated in clinical trials for the treatment of various diseases [32], [33]. Reported STAT5 inhibitors target STAT5 activity at multiple levels, from its activation by phosphorylation to its DNA binding activity, independently of upstream activating kinases [30], [34], [35], [38], [39], [45]. We were the first to show that deacetylase inhibitors such as sodium butyrate, trichostatin A (TSA) or suberoylanilide hydroxamic acid (SAHA) inhibit IL-3-mediated STAT5 transcriptional activity in the mouse pro-B cell line Ba/F3 [46]. We demonstrated that deacetylase inhibitors interfere neither with STAT5 initial activation (phosphorylation or nuclear translocation) nor with STAT5 binding to DNA *in vivo*. Instead, these drugs prevent the proper assembly of the basal transcription machinery ultimately resulting in inhibition of transcription [46], [47]. The deacetylase inhibitor SAHA (Vorinostat) has been approved for the treatment of cutaneous T cell lymphoma while many other deacetylase inhibitors are currently evaluated in clinical trials for the treatment of various types of cancers [48]. Therefore our findings support the attractive possibility that this class of drugs might be efficient in targeting STAT5-associated cancers. Accordingly, tyrosine kinase inhibitor and deacetylase inhibitor combination therapies have recently proven to be more effective in the treatment of cancers with constitutive active STAT5 [49]–[53]. More recently, we turned to the identification of new types of STAT5 inhibitors, with a particular focus on natural and synthetic chalcones.

**Figure 1:**
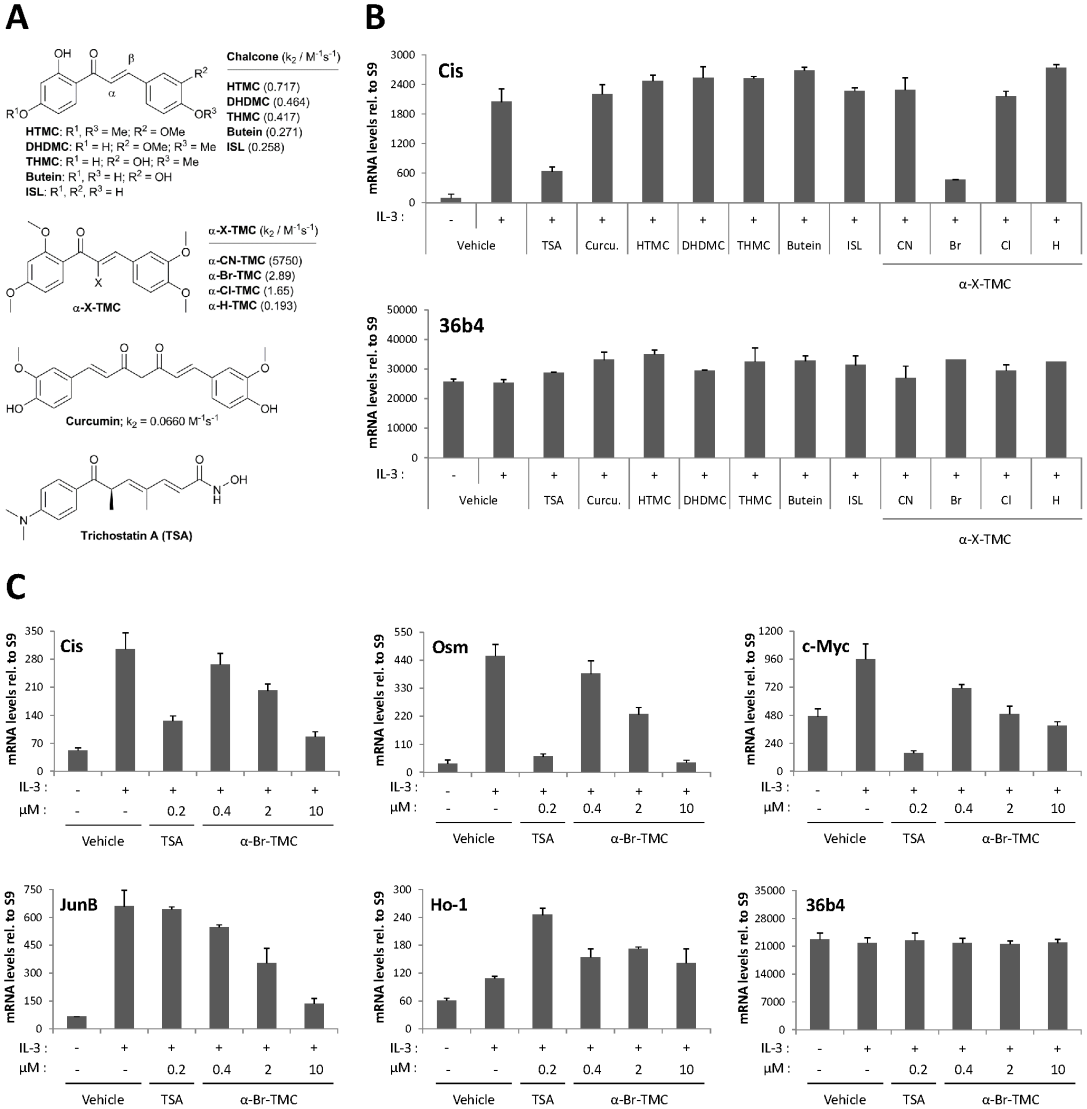
α-Br-TMC inhibits IL-3-mediated induction of STAT5-dependent and -independent genes in Ba/F3 cells. (**A**) Structure of the natural and synthetic chalcones used in this study. Second-order rate constants k_2_ values of compounds obtained using cysteamine in TRIS-HCl buffer pH 7.4: ethylene glycol 20:80 are taken from refs. [59], [60]. (**B**) Ba/F3 cells were pre-treated 30 minutes with 0.2 µM (TSA) or 20 µM (all other compounds) candidate inhibitors and stimulated 60 minutes with 5 ng/mL IL-3, as described in Materials and Methods. Following cell harvest, expression of the STAT5 target gene *Cis* and of the housekeeping gene *36b4* were measured by quantitative RT-PCR, as described in Materials and Methods. Together with TSA, α-Br-TMC was the only compound able to inhibit expression of the STAT5 target gene *Cis*. (**C**) Ba/F3 cells were pre-treated 30 minutes with the indicated concentrations of TSA and α-Br-TMC and further stimulated with 5 ng/mL IL-3 for 30 minutes. Expression of STAT5-dependent (*Cis*, *Osm*, *c-Myc*) and -independent (*JunB*, *Ho-1*, *36b4*) genes was analyzed by quantitative RT-PCR. DMSO (vehicle) was adjusted to 0.02% final concentration in all conditions. Curcu., curcumin.

Chalcones, which are naturally abundant in fruits, vegetables and spices, are α,β-unsaturated carbonyl compounds well known for their multiple biological activities as antioxidant, antibacterial, antifungal, anti-inflammatory and anticancer agents [54]. Natural and synthetic chalcones have been shown to inhibit the function of a variety of tyrosine kinases (JAKs, ERKs, Src) and transcription factors (NF-κB, STAT3) implicated in the response to inflammation and the control of cell proliferation and survival [54]–[58]. We therefore hypothesized that the JAK2/STAT5 signaling pathway might be affected by chalcones. We previously described the synthesis of α-X-2′,3,4,4′-tetramethoxychalcones (α-X-TMCs) with different X substituents (X = H, F, Cl, Br, I, CN, Me, *p*-NO_2_-C_6_H_4_, Ph, *p*-OMe-C_6_H_4_, NO_2_, CF_3_, COOEt, COOH) at the α-position of the α,β-unsaturated carbonyl unit [59]. We formerly showed that these α-X-TMCs have distinct electrophilic reactivities [59]–[61] which correlate well with their anti-inflammatory activity [59]. In this study, the ability of four α-X-TMCs (with X = CN, Br, Cl, H) to regulate STAT5 function was investigated. Five natural chalcones, namely 2′-hydroxy-3,4,4′-trimethoxychalcone (HTMC), 2′,4′-dihydroxy-3,4-dimethoxychalcone (DHDMC), 2′,3,4′-trihydroxy-4-methoxychalcone (THMC), butein, and isoliquiritigenin (ISL), as well as curcumin, another natural product carrying two α,β-unsaturated carbonyl groups, were also investigated (Figure 1A).

We describe here α-Br-2′,3,4,4′-tetramethoxychalcone (α-Br-TMC) as a novel inhibitor of the STAT5 signaling pathway. We show that α-Br-TMC inhibits both JAK2 and STAT5 phosphorylation, leading to the inhibition of expression of downstream STAT5-dependent and -independent genes in IL-3-stimulated Ba/F3 cells. Moreover, α-Br-TMC alters the mobility of STAT5A and STAT5B proteins in SDS-PAGE, both in cells expressing regulated (Ba/F3) and constitutively active (Ba/F3-1*6 and K562) STAT5, presumably reflecting a change in STAT5 post-translational modifications. Nevertheless, α-Br-TMC treatment affects differentially STAT5 transcriptional activity in normal and cancer cells. Altogether, this study identified a novel inhibitor of STAT5 signaling targeting both STAT5 and its upstream activating kinase JAK2, and affecting the regulation of downstream oncogenes and tumor suppressor genes.

## Materials and Methods

### Chemicals

Chalcones were prepared by published procedures [59], [60]. Dimethyl sulfoxide (DMSO), trichostatin A (TSA) and curcumin were purchased from SIGMA (D2650, T8552 and B6938 respectively). Imatinib was from Cayman Chemical (No. 13139). Compounds were dissolved in DMSO at a final concentration of 1 mM (TSA) or 100 mM (other compounds).

### Thiol assay

Kinetic thiol assay for TSA (Biotrend BN0742) was performed as described [60] using 500-fold excess cysteamine as a thiol substrate and an incubation time of 48 hours. The presence of the addition product was detected by liquid chromatography-mass spectrometry (not shown). Second-order rate constants k_2_ values for chalcones and curcumin are taken from the literature [59], [60].

### Cell lines and drug treatments

All cell lines were grown in RPMI 1640 (PAN-Biotech P04-16500) supplemented with 10% heat-inactivated fetal calf serum (FCS; PAN-Biotech), penicillin/streptomycin (PAN-Biotech) (thereafter designated as RPMI-based medium) and cultivated at 37°C under 5% CO_2_ in a humidified incubator. K562 cells (a kind gift from Daniela Männel, University of Regensburg, Germany; [62]) were maintained in RPMI-based medium. The non-tumorigenic immortalized interleukin-3 (IL-3)-dependent mouse pro-B cell line Ba/F3 (a kind gift from Jacqueline Marvel, IFR 128 BioSciences Gerland-Lyon Sud, France; [63]) was grown in RPMI-based medium supplemented with 2 ng/mL rmIL-3 (ImmunoTools). The Ba/F3-1*6 cell line stably expressing the constitutively active mouse STAT5A-1*6 mutant [64] was generated according to German GenTSV (genetic engineering safety regulations; authorization AZ.55.1-8791.7.52) by electroporating Ba/F3 cells with a pcDNA3-based expression vector allowing expression of a mSTAT5A-1*6-FLAG fusion protein. Stably transfected cells were selected in IL-3-free medium in the presence of 500 µg/mL G418 (PAA). Individual IL-3-independent clones were isolated and characterized to verify mSTAT5A-1*6 transgene expression and constitutive activity, as originally described [64]. Clone Ba/F3-1*6 F7 was used for this study. Cells were maintained in RPMI-based medium supplemented with 500 µg/mL G418.

For cytokine stimulation of Ba/F3 cells, cells were washed twice in RPMI 1640 and rested in RPMI-based medium for 12 hours before addition of 5 ng/mL IL-3. Duration of IL-3 stimulation was contingent upon the downstream assay, as indicated in the figure legends. For chalcone and other inhibitor treatments, Ba/F3 cells were incubated with compounds or DMSO (vehicle) for 30 minutes prior to cytokine stimulation. Ba/F3-1*6 and K562 cells were treated for 60–90 minutes with the various compounds or DMSO (vehicle), as indicated. With the exception of cell viability assays (see below), all experiments were performed in the presence of equal amounts of DMSO.

### Gene expression analysis by quantitative RT-PCR

Following inhibitor and cytokine treatments, cells were harvested, lysed in the iScript RT-qPCR sample preparation reagent (170–8899, Bio-Rad Laboratories) and subjected to cDNA synthesis using the iScript cDNA Synthesis kit (170–8891, Bio-Rad Laboratories), following the manufacturer's instructions. Quantitative PCR was performed on a RotorGene Q (Qiagen) with SYBR Green I and HotStarTaq (Qiagen). Data were normalized to either S9 ribosomal mRNA (Ba/F3 and Ba/F3-1*6 cell lines) or human Lamin A/C (LMNA) mRNA (K562 cell line), and expressed as relative mRNA levels, as previously described [3], [46], [47]. Real-time PCR primers specific for the following mouse (m) or human (h) cDNAs have been described: *S9* (m), *Cis* (m), *c-Myc* (m/h), *Pim-1* (m), *Osm* (m), *c-Fos* (m), *Jun-B* (m), *36b4* (m) and LMNA (h) [46], [65]. Forward (fwd) and reverse (rev) real-time PCR primers specific for human *Cis* cDNA were 5′- CTGCTGTGCATAGCCAAGACC-3′ (fwd) and 5′-GTGCCTTCTGGCATCTTCTGC-3′ (rev), and for mouse *Ho-1* cDNA were 5′-GCTGGTGATGGCTTCCTTGT-3′ (fwd) and 5′-GGTTCTGCTTGTTGCGCTCT-3′ (rev). Data shown are representative of at least three independent experiments.

### Cytotoxicity assays

WST-1 assays (11 644 807 001, Roche) were performed as described in the manufacturer's protocol. The tetrazolium salt WST-1 is cleaved into the formazan dye by mitochondrial dehydrogenase enzymes. Changes in metabolically active mitochondrial dehydrogenases as a result of TSA- or α-Br-TMC-induced cytotoxicity was evaluated by measuring formazan dye production upon incubation with the WST-1 reagent. Rested Ba/F3 and growing Ba/F3-1*6 and K562 cells were pre-treated for 30 minutes with the indicated TSA and α-Br-TMC concentrations or with DMSO (vehicle), as in our gene expression analysis experiments. WST-1 reagent was added to the cells either alone (Ba/F3-1*6, K562) or together with IL-3 (Ba/F3). Absorbance was measured in a microplate reader (Mithras LB 940, Berthold Technologies) at 450/620 nm after incubation at 37°C under 5% CO_2_ in a humidified incubator for 90 minutes. This duration of treatment was chosen as it is both optimal for the WST-1 assay (not shown) and close to the maximal duration of inhibitor treatment in our gene expression studies. A positive control for no mitochondrial enzyme activity (1% Triton X-100) was included in every experiment. Data are expressed as a percentage of cytotoxicity relative to DMSO (vehicle). Small molecule inhibitor treatment was carried out 2 to 3 times for each cell line, and the WST-1 assay was performed in duplicate. Results of one representative experiment are shown.

### Cell viability assays

Equal cell numbers of growing Ba/F3, Ba/F3-1*6 and K562 cells were incubated for 24 and 48 hours in the presence of the indicated TSA and α-Br-TMC concentrations. Since very low DMSO concentrations were used (0.0005% to 0.01% final), no DMSO vehicle control was included. The total number of living and dead cells was evaluated by Trypan Blue exclusion. Viable cell number, reflecting cell proliferation and survival, was plotted as a function of time for each treatment. Data shown are representative of at least three independent experiments.

### Quantitative chromatin immunoprecipitation (ChIP) assays

Chromatin immunoprecipitation was performed as previously described [3], [46], [47] with the following modifications: sonication was performed on a BRANSON SONIFIER 250 and cell samples were subjected to 6 pulses of 60 seconds each (output control: 5; duty cycle: 60%); Immunoprecipitated genomic DNA was purified on a Nucleospin clean-up column (740609, Macherey-Nagel) following their recommended protocol for SDS-containing samples. STAT5 and RNA polymerase II antibodies, as well as real-time PCR primers specific for the STAT5 binding sites of the mouse *Cis* gene and for the transcription start site (tss) of the mouse *Cis* and *Osm* genes, have been described [46]. Real-time PCR primers specific for the mouse *Osm* STAT5 binding sites (−184/−122 relative to tss) were 5′-CATCATCCTTGGGCGTGGGGC-3′ (fwd) and 5′-CGCTCCTCCTCCCGTTTTCTTCG-3′ (rev). Data shown are representative of at least three independent experiments.

### Protein analysis

Whole-cell Brij protein lysis, nuclear and cytosolic protein extractions, and Western-blot analyses were performed as described [46], [65]. Antibodies used for detection of the respective proteins and their relevant dilutions were: pSTAT5 (#9351, Cell Signaling Technology; 1∶1000), STAT5A (L-20, sc-1081, Santa-Cruz Biotechnology; 1∶1000), STAT5B (G-2, sc-1656, Santa-Cruz Biotechnology; 1∶200), STAT5A+B (C-17, sc-835, Santa-Cruz Biotechnology; 1∶1000), pJAK2 (Cell Signaling Technology, #3771; 1∶200), JAK2 (#3230, Cell Signaling Technology; 1∶500), α-tubulin (DM1A, sc-32293, Santa-Cruz Biotechnology; 1∶200), Anti-Rabbit IgG-Peroxidase (SIGMA A0545; 1∶10,000), Anti-Mouse IgG-Peroxidase (SIGMA A8924; 1∶10,000). Apparent molecular weight of detected proteins was as predicted by the antibody manufacturers, i.e. 125 kDa for JAK2, 90 kDa for STAT5 (with STAT5A running slightly slower than STAT5B in SDS-PAGE, as confirmed by the STAT5A+B immunoblots) and 55 kDa for α-tubulin. Chemoluminescence detection was performed using Amersham ECL Prime (RPN2232, GE Healthcare Life Sciences) or SuperSignal West Femto (34095, Thermo Fisher Scientific) for weaker signals, and images were captured on an ImageQuant LAS 4000 mini imaging system (GE Healthcare Life Sciences). Immunoblots shown are representative of at least three independent experiments.

## Results

### The α-Br-chalcone α-Br-TMC inhibits expression of IL-3-induced STAT5-dependent and -independent genes in mouse Ba/F3 cells

In our initial screening for potential STAT5 inhibitors we chose a variety of natural and synthetic chalcones characterized towards their chemical reactivity in Michael additions with thiols. The respective second-order rate constants (k_2_) of α-X-TMCs (with X = CN, Br, Cl, H) using cysteamine as substrate [59]–[61] revealed differences of more than four orders of magnitude (Figure 1A), pointing to large variations in electrophilic reactivity. We also included the natural product curcumin, which is a weak electrophile (k_2_ = 0.0660 M^−1^s^−1^) [60], and the synthetic lysine deacetylase inhibitor trichostatin A (TSA), which serves as a reference compound for STAT5 inhibition [46], [47]. A kinetic thiol assay on TSA, which contains an α,β,γ,δ-unsaturated carbonyl unit, revealed very low amounts of the expected thiol adduct by mass spectrometry after 48 hours incubation with 500-fold excess of cysteamine (data not shown). This indicates that TSA main activity is not based on an electrophilic behavior, as anticipated from its electron-rich carbonyl functionality.

To identify novel inhibitors of the STAT5 signaling pathway, these ten natural and synthetic chalcones as well as curcumin were tested for their ability to suppress IL-3-mediated induction of the STAT5 target gene *Cis*. The gene *Cis* was selected for the initial screen as it is a well characterized STAT5-dependent gene [3], [46], [47], [66] and a potential therapeutic target in STAT5-associated cancers [67].

Ba/F3 cells withdrawn from IL-3 for 12 hours (STAT5 pathway turned off) were pre-treated for 30 minutes with 0.2 µM TSA or with 20 µM natural and synthetic chalcones or curcumin, before being stimulated with IL-3 for 1 hour (Figure 1B). Out of the 10 compounds tested, one (α-Br-2′,3,4,4′-tetramethoxychalcone thereafter called α-Br-TMC) exerted an 80% inhibition of IL-3-mediated induction of *Cis* expression, comparable to that exerted by TSA, while the housekeeping gene *36b4* remained unaffected (Figure 1B). In the following experiments, we therefore focused on characterizing α-Br-TMC activity, using TSA as a reference inhibitory compound.

**Figure 2:**
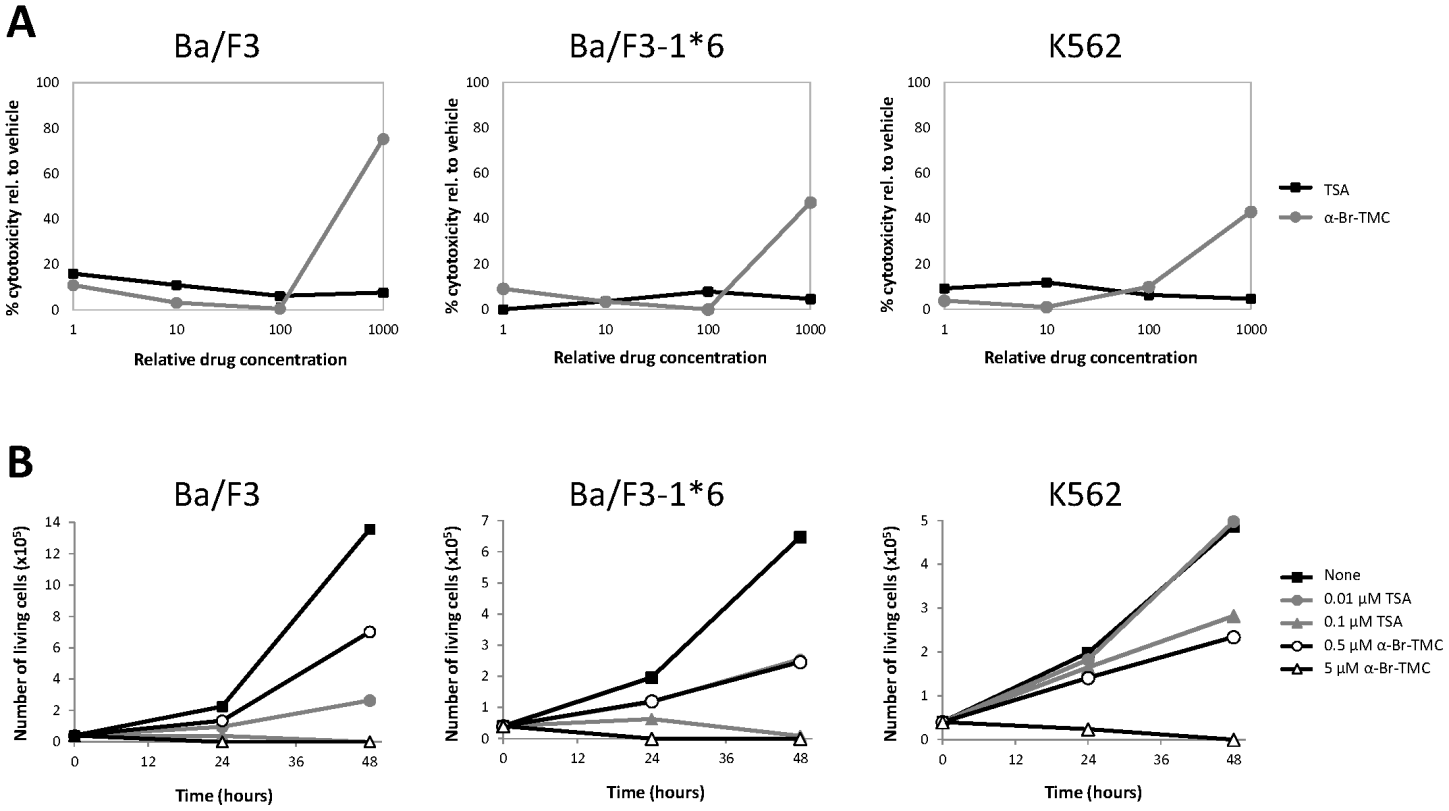
Effect of α-Br-TMC on cytotoxicity and viability of normal (Ba/F3) and transformed (Ba/F3-1*6, K562) cells. (**A**) Cells were pre-treated 30 minutes with 0.001, 0.01, 0.1 and 1 µM TSA or with 0.1, 1, 10 and 100 µM α-Br-TMC before starting the WST-1 assay. IL-3 (5 ng/mL) was added to rested Ba/F3 cells at the same time as the WST-1 reagent to mimic the IL-3 stimulation conditions used in other assays. DMSO (vehicle) concentration was adjusted to 0.1% final in all conditions. OD measurement was performed after 90 minutes incubation with the WST-1 reagent, and the percentage of cytotoxicity was normalized to the vehicle control. (**B**) Growing Ba/F3, Ba/F3-1*6 and K562 cells were incubated for 24 and 48 hours in the presence of the indicated concentrations of TSA and α-Br-TMC. Cell viability was measured by Trypan Blue exclusion assay.

First, we ensured the absence of α-Br-TMC-mediated cytotoxicity in the investigated time frame (up to 90 minutes treatment) in IL-3-stimulated Ba/F3 cells (Figure 2A). No cytotoxicity was detected up to 10 µM α-Br-TMC. However, strong cytotoxicity was evidenced at an α-Br-TMC concentration of 100 µM (Figure 2A). Thus α-Br-TMC was further used at concentrations not above 10 µM. We also investigated the long term effect of α-Br-TMC treatment on cell proliferation and survival of IL-3-growing Ba/F3 cells (Figure 2B). Similarly to TSA, α-Br-TMC impaired cell growth and viability in a dose-dependent manner. Ba/F3 cells cultured in 5 µM α-Br-TMC for 24 and 48 hours stopped dividing and died, while cells grown in 0.5 µM α-Br-TMC showed limited cell death and reduced cell proliferation (Figure 2B and data not shown).

We then analyzed expression of a series of STAT5 target genes (*Cis*, *Osm*, *c-Myc*) as well as STAT5-independent genes (*JunB*, *Ho-1*, *36b4*) in dose-response experiments using 0.4 to 10 µM α-Br-TMC. Rested Ba/F3 cells were incubated 30 minutes with the respective compounds and stimulated with IL-3 for 30 minutes (Figure 1C). Induction of all three STAT5 target genes was inhibited by α-Br-TMC in a dose-dependent manner. In addition, the IL-3-inducible STAT5-independent gene *JunB*, while unaffected by TSA as expected [46], was also inhibited by α-Br-TMC in a dose-dependent manner (Figure 1C). It is thought that *JunB* is induced through the JAK2/MAPK pathway [68], [69], therefore suggesting that α-Br-TMC is targeting the upstream JAK/STAT pathway rather than STAT5 itself. α-Br-TMC was shown to up-regulate the Nrf2-dependent anti-inflammatory HO-1 protein levels in the mouse macrophage cell line RAW264.7 [59]. In Ba/F3 cells however, although *Ho-1* gene expression was slightly up-regulated by IL-3 and TSA, no dose-dependent effect of α-Br-TMC was observed (Figure 1C), suggesting that the Nrf2-dependent anti-inflammatory response pathway is - if at all - only modestly activated and not influenced by α-Br-TMC. In contrast to IL-3-regulated genes, expression of the control gene *36b4* remained unaffected by α-Br-TMC treatment (Figure 1C), further supporting specificity of action.

Together, these gene expression data suggest that α-Br-TMC might target the JAK/STAT pathway for inhibition. We therefore monitored the effect of α-Br-TMC on STAT5 and JAK2 protein phosphorylation.

### α-Br-TMC inhibits both JAK2 and STAT5 phosphorylation

**Figure 3:**
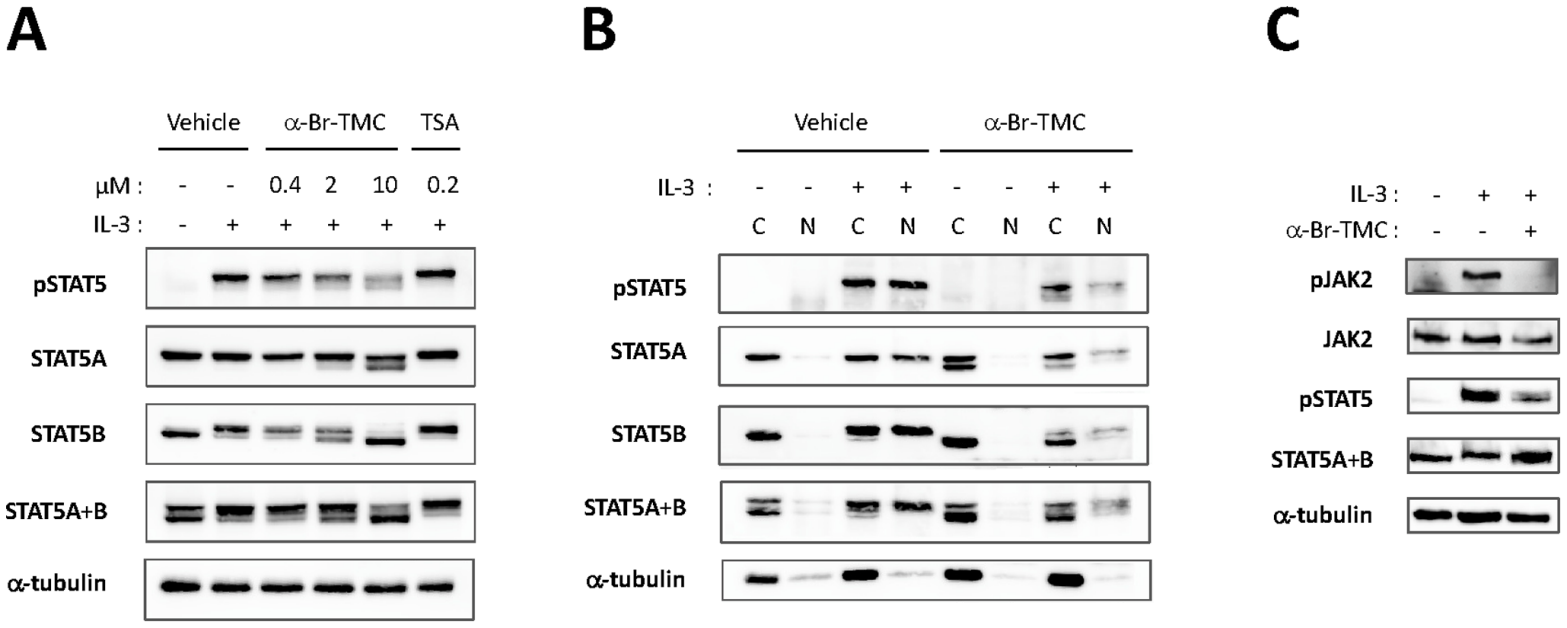
α-Br-TMC inhibits both JAK2 and STAT5 phosphorylation. Ba/F3 cells were pre-treated 30 minutes with the indicated concentrations of TSA and α-Br-TMC (**A**) or with 10 µM α-Br-TMC and DMSO vehicle (**B, C**) and further stimulated with 5 ng/mL IL-3 for 30 minutes (**A**), 15 minutes (**B**) or 5 minutes (**C**). Final DMSO concentration was 0.02% in (**A**) and 0.01% in (**B, C**). Protein whole cell extracts (**panels A, C**) or cytosolic (C) and nuclear (N) extracts (**panel B**) were prepared as described in Materials and Methods and analyzed by Western-blot using antibodies specific for phospho-STAT5 (pSTAT5), phospho-JAK2 (pJAK2), STAT5A, STAT5B, STAT5A and B, JAK2 and α-tubulin (loading control and cytosolic-specific marker). SDS-PAGE in (C) was shorter, explaining why the STAT5 mobility patterns (α-Br-TMC-induced shift and STAT5A and B doublet) are not as apparent as on immunoblots in (A, B).

STAT5 proteins are expressed in two forms, STAT5A and STAT5B, with both redundant and unique functions and encoded by two related genes. STAT5A and STAT5B exhibit 91% identity in their amino acid sequence, the C-terminal transactivation domain being most divergent [3]. STAT5 phosphorylation in IL-3-stimulated Ba/F3 cells pre-treated with either 0.2 µM TSA or 0.4 to 10 µM α-Br-TMC was investigated by Western-blot using a phospho-STAT5-specific antibody (pSTAT5) (Figure 3A). IL-3-induced STAT5 phosphorylation was not affected by TSA, in agreement with our previous report [46]. By contrast, IL-3-induced STAT5 phosphorylation was decreased by α-Br-TMC in a dose-dependent manner. Interestingly, both STAT5A and STAT5B proteins showed a dose-dependent downward mobility shift in SDS-PAGE, with STAT5B being more affected than STAT5A (Figure 3A). This mobility shift suggests that α-Br-TMC induces a protein modification targeting both STAT5 proteins. Analysis of cytosolic and nuclear fractions of unstimulated and stimulated α-Br-TMC-treated cells, revealed that this α-Br-TMC-mediated protein modification is taking place in the cytoplasm of unstimulated cells (Figure 3B) and hence is IL-3-independent. Analysis of cytosolic and nuclear fractions of α-Br-TMC-treated cells also confirmed that STAT5 phosphorylation and nuclear translocation are reduced (Figure 3B), thus supporting the idea that STAT5 activation is impaired upon α-Br-TMC treatment. Of note, the detection of shifted bands by the pSTAT5 antibody in the cytosolic and nuclear fractions of α-Br-TMC-treated IL-3-stimulated cells, suggests that the modified STAT5 protein can be to some extent phosphorylated at the expected tyrosine residue (Y694/699 in STAT5A/B respectively) and translocated into the nucleus.

STAT5 activation by IL-3 relies on the initial activation of the cellular tyrosine kinase JAK2 [70]. Our gene expression data revealing an inhibitory effect of α-Br-TMC on expression of the JAK2/MAPK-dependent *JunB* gene (Figure 1C) suggest that α-Br-TMC might also target the upstream JAK2 tyrosine kinase. We therefore investigated whether IL-3-induced phosphorylation of JAK2 was inhibited by α-Br-TMC. Ba/F3 cells were treated as before with 10 µM α-Br-TMC and briefly stimulated with IL-3 to analyze JAK2 phosphorylation by Western-blot using a pJAK2-specific antibody (Figure 3C). Following α-Br-TMC treatment, JAK2 phosphorylation was strongly diminished. By contrast to STAT5A and STAT5B proteins, JAK2 protein mobility was not affected by α-Br-TMC (Figure 3C).

Altogether, these experiments demonstrated that the α-Br-chalcone α-Br-TMC has the ability to inhibit the JAK/STAT pathway at multiple levels, by targeting both JAK2 and STAT5 proteins in IL-3-stimulated Ba/F3 cells.

### α-Br-TMC inhibits STAT5-mediated transcriptional activity

**Figure 4:**
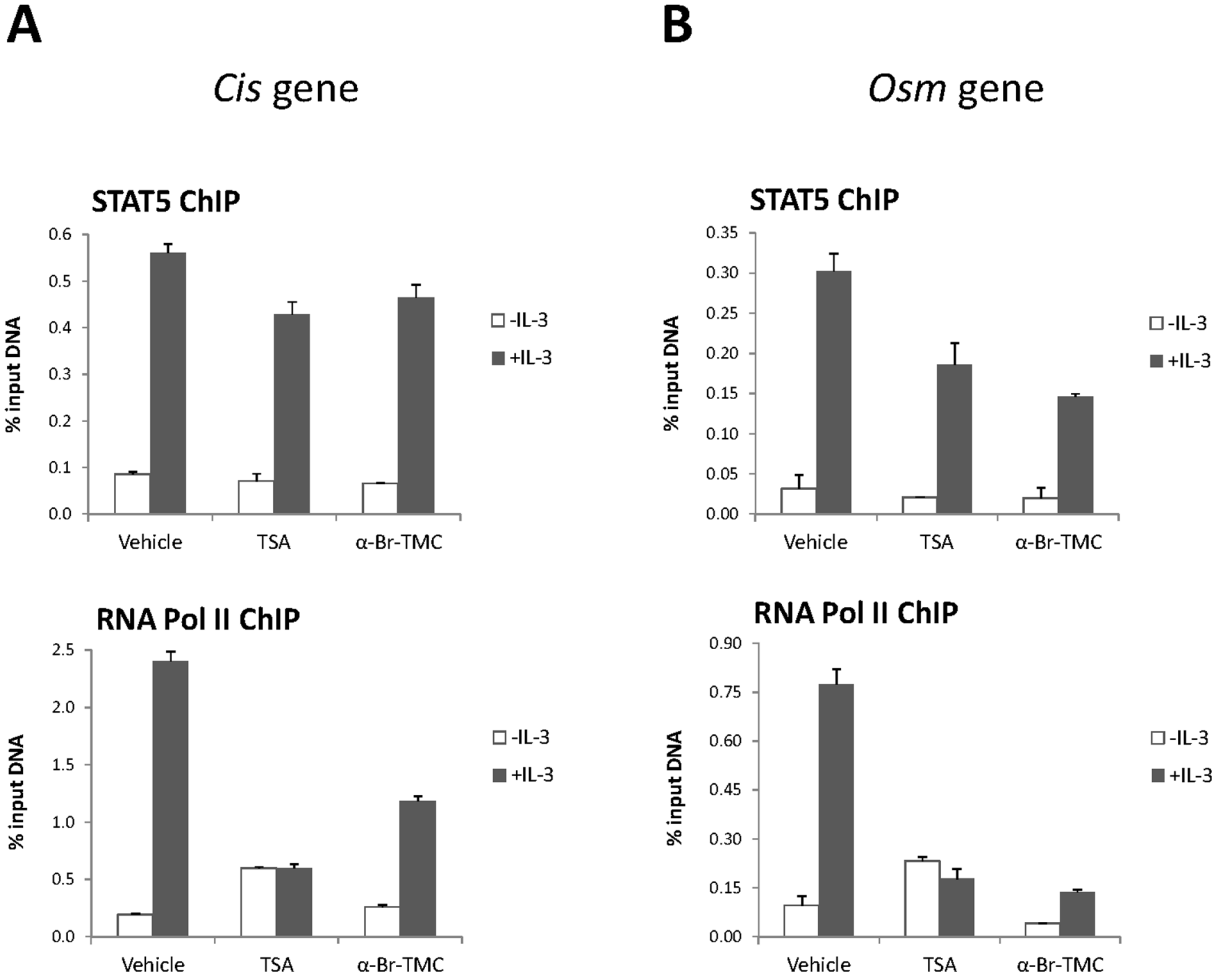
RNA polymerase II recruitment to the *Cis* and *Osm* promoters is impeded in Ba/F3 cells treated with α-Br-TMC. Ba/F3 cells were pre-treated 30 minutes with 0.2 µM TSA or 10 µM α-Br-TMC before being stimulated with 5 ng/mL IL-3 for 30 minutes. DMSO (vehicle) was adjusted to 0.02% in all conditions. Chromatin immunoprecipitation (ChIP) was performed as described in Materials and Methods using antibodies directed against STAT5 or RNA polymerase II (RNA Pol II) proteins. Co-precipitated genomic DNA was analyzed by quantitative PCR using primers specific for the STAT5 binding sites (STAT5 ChIP) or the transcription start site (RNA Pol II ChIP) of the mouse *Cis* (**A**) and *Osm* (**B**) genes.

The effect of α-Br-TMC on STAT5 transcriptional activity was further investigated by chromatin immunoprecipitation (ChIP). We previously showed that the deacetylase inhibitor TSA inhibits STAT5-mediated transcription at a step subsequent to STAT5 binding to its target genes by preventing recruitment of the transcriptional machinery [46]. STAT5 and RNA polymerase II ChIP assays were performed on TSA- and α-Br-TMC-treated Ba/F3 cells and protein recruitment to the mouse *Cis* and *Osm* promoters was examined (Figure 4). In agreement with our former report [46], RNA polymerase II recruitment to the *Cis* and *Osm* promoters was abrogated upon TSA treatment, without notably affecting STAT5 DNA binding activity. Although the level of nuclear pSTAT5 was strongly reduced in α-Br-TMC-treated cells (Figure 3B), the proportion of STAT5 bound to the *Cis* and *Osm* promoters was only moderately affected (20% and 58% reduced respectively; Figure 4A and B). This indicates that the nuclear pool of STAT5 proteins in α-Br-TMC-treated Ba/F3 cells might still support efficient binding to target sites. On the other hand, in correlation with the reduced Cis and Osm mRNA levels (Figure 1), recruitment of the RNA polymerase II to the *Cis* and *Osm* transcription start sites was markedly impaired (55% and 94% respectively; Figure 4A and B), pointing to an α-Br-TMC-mediated transcriptional inhibition of the *Cis* and *Osm* STAT5 target genes in Ba/F3 cells.

### α-Br-TMC differentially affects JAK/STAT signaling in cells transformed by constitutively active STAT5

We showed that α-Br-TMC can inhibit IL-3-mediated JAK2 and STAT5 activation in normal mouse Ba/F3 cells. We next addressed whether α-Br-TMC can also hinder JAK/STAT activity in cancer cells exhibiting constitutive activation of STAT5. For this purpose, we compared gene expression patterns of normal Ba/F3 cells to those of transformed Ba/F3-1*6 and K562 cells.

Ba/F3-1*6 cells stably express a mutant form of mouse STAT5A (so-called 1*6) with two amino acids substitutions within its N-terminal and C-terminal transactivation domains respectively. These point mutations result in constitutive STAT5 phosphorylation, nuclear localization and transactivation properties [64]. Expression of constitutively active STAT5A-1*6 is sufficient to confer IL-3-independent growth to Ba/F3 cells *in vitro* and tumorigenicity to bone marrow cells *in vivo* [64], [71]. Importantly, JAK2 is not activated in Ba/F3-1*6 cells as a consequence of IL-3-independent growth [4], [64]. The mechanism of constitutive activation of STAT5A-1*6 is unclear. Whether basal JAK2 activity or an unidentified tyrosine kinase is responsible for STAT5A-1*6 phosphorylation is unknown [4], [64]. Increased stability of the phosphorylated STAT5A-1*6 mutant via an uncharacterized mechanism also contributes to its constitutive activity [64]. The human K562 leukemia cell line expresses a constitutively active BCR-ABL tyrosine kinase. BCR-ABL oncogenic fusion protein constitutively phosphorylates STAT5A and STAT5B proteins, directly contributing to oncogenesis [72]–[74].

**Figure 5:**
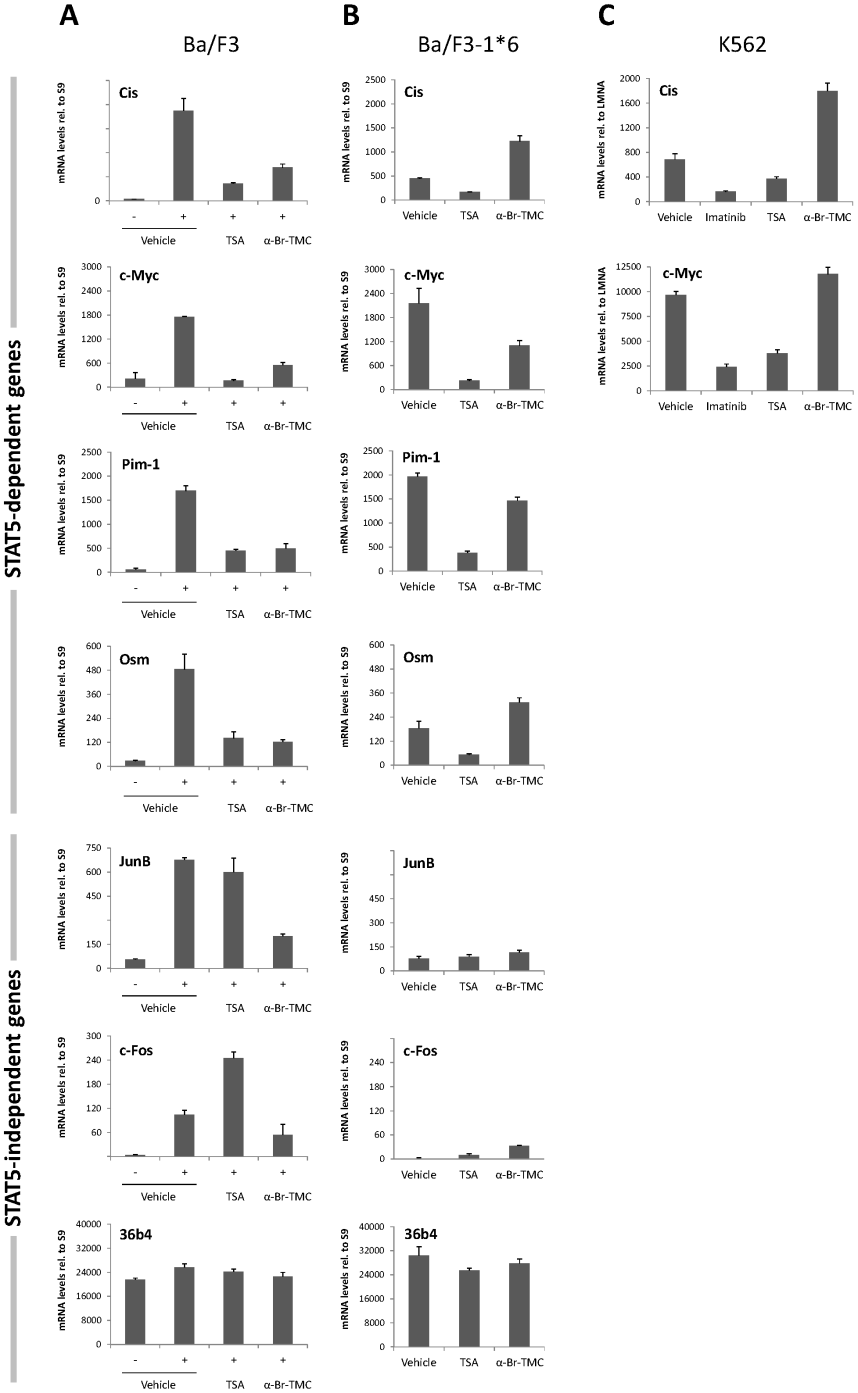
α-Br-TMC exerts distinct effects in normal and cancer cells. Ba/F3 (**A**), its caSTAT5-transformed counterpart Ba/F3-1*6 (**B**) and human leukemic K562 (**C**) cells were treated 90 minutes with 0.2 µM TSA, 10 µM α-Br-TMC or 1 µM Imatinib. Ba/F3 cells (**A**) were stimulated with 5 ng/mL IL-3 after an initial 30 minute drug pre-treatment (hence subjected to a 60 minute IL-3 stimulation). DMSO (vehicle) final concentration was adjusted to 0.02% in all conditions. Expression of STAT5-dependent (*Cis*, *Osm*, *c-Myc, Pim-1*) and -independent (*JunB*, *c-Fos*, *36b4*) genes was analyzed by quantitative RT-PCR. Gene expression data were normalized to mouse ribosomal *S9* (**A, B**) or to human Lamin A/C (*LMNA*) (**C**) housekeeping gene-encoded mRNAs. (**A, B**) Normalized data are presented with adjusted Y-axis scale for a direct comparison of mRNA levels in the respective normal and transformed Ba/F3 and Ba/F3-1*6 cell lines.

Ba/F3, Ba/F3-1*6 and K562 cells were treated with 0.2 µM TSA or 10 µM α-Br-TMC for 90 minutes, with Ba/F3 being stimulated 60 minutes with IL-3 following 30 minute inhibitor pre-treatment, as before. In addition, K562 cells were treated with 1 µM of the BCR-ABL specific inhibitor Imatinib, as a positive control for inhibition of STAT5 phosphorylation [75]. Expression of STAT5 target genes (*Cis*, *Osm*, *c-Myc, Pim-1*), of JAK2/MAPK-regulated STAT5-independent genes (*JunB*, *c-Fos*), and of a housekeeping control gene (*36b4*) was evaluated by quantitative RT-PCR (Figure 5). As predicted upon expression of constitutively active STAT5-1*6 [46], expression of the STAT5 target genes was up-regulated in growing Ba/F3-1*6 cells in comparison to unstimulated Ba/F3 cells (Figure 5A–B). As anticipated, expression of the STAT5 target genes in all three cell lines was inhibited by TSA (Figure 5A–C). Similarly, STAT5 target genes were down-regulated in Imatinib-treated K562 cells (Figure 5C). Expression of all STAT5-target genes investigated in IL-3-stimulated Ba/F3 cells, including *Cis*, *Osm*, *c-Myc, Pim-1* (Figure 5A) as well as *Spi2.1* and *SOCS-1* (not shown) was inhibited by α-Br-TMC. While expression of STAT5 target genes was down-regulated in α-Br-TMC-treated Ba/F3 cells (Figure 5A), distinct effects of α-Br-TMC were noted in Ba/F3-1*6 and K562 cells (Figure 5B–C). For instance, expression of *Cis* and *Osm* was up-regulated in α-Br-TMC-treated Ba/F3-1*6 (Figure 5B) and K562 (Figure 5C) cells. c-Myc and Pim-1 mRNA levels were partially reduced in α-Br-TMC-treated Ba/F3-1*6 cells (Figure 5B), while c-Myc mRNA level was slightly increased in α-Br-TMC-treated K562 cells (Figure 5C). Therefore, the α-Br-2′,3,4,4′-tetramethoxychalcone exerts distinct effects on STAT5 target gene expression in normal Ba/F3 vs. its transformed counterpart Ba/F3-1*6, as well as in the oncogenic K562 cell line.

Both JAK2-mediated MAPK-dependent genes *JunB* and *c-Fos* were induced by IL-3 in Ba/F3 cells (Figure 5A). Their expression was either unaffected or upregulated by TSA respectively, as previously reported [46]. Expression of *JunB* and *c-Fos* was inhibited by α-Br-TMC, probably as a consequence of JAK2 inhibition (Figure 5A). In contrast to Ba/F3 cells, *JunB* and *c-Fos* genes were expressed at background levels in Ba/F3-1*6 cells (Figure 5B), most likely as a result of the absence of JAK2/MAPK activation [4]. Treatment of Ba/F3-1*6 cells with α-Br-TMC did not affect *JunB* expression while *c-Fos* was slightly up-regulated (Figure 5B), although mRNA levels remained low in comparison to that detected in IL-3-stimulated Ba/F3 cells (Figure 5A). The observation that α-Br-TMC was able to up-regulate the expression of *c-Fos* - and of other genes - in Ba/F3 cells transformed with STAT5A-1*6 but not in Ba/F3 parental cells, suggests that α-Br-TMC might target other factor(s) involved in gene regulation in Ba/F3-1*6 cells.

To further evaluate the effect of α-Br-TMC on normal and transformed cells, cytotoxicity and cell viability assays were performed as before (Figure 2A–B). The effect of α-Br-TMC on proliferation and survival of Ba/F3-1*6 and K562 cells was comparable to that observed with normal Ba/F3 cells. All cells died in the presence of 5 µM α-Br-TMC, while reduced proliferation and minimal cell death were observed in the presence of 0.5 µM α-Br-TMC over 48 hours (Figure 2B and data not shown). On the other hand, α-Br-TMC-induced cytotoxicity (at 100 µM) was reduced by approximately 50% in the transformed Ba/F3-1*6 and K562 cell lines in comparison to normal Ba/F3 cells (Figure 2A; 75% cytotoxicity in Ba/F3 cells vs. 47% and 42% in Ba/F3-1*6 and K562 cells respectively). This indicates a distinct chemosensitivity of the cancer cell lines to α-Br-TMC. Together with the observation that α-Br-TMC differentially affects gene expression in the transformed cells, our results suggest that its activity is regulated in a distinct manner in the investigated normal vs. transformed cell lines.

In conclusion, our gene expression analysis demonstrated that α-Br-TMC differentially regulates expression of STAT5 target genes in normal and cancer cells. Since we showed that α-Br-TMC hinders STAT5 transcriptional activity in Ba/F3 cells by inhibiting both STAT5 and JAK2 phosphorylation and possibly by modifying STAT5 proteins, we examined the effect of α-Br-TMC on JAK2 and STAT5 proteins in the transformed Ba/F3-1*6 and K562 cells.

### α-Br-TMC-induced STAT5 protein modification differentially modulates STAT5 activity in normal and cancer cells

**Figure 6:**
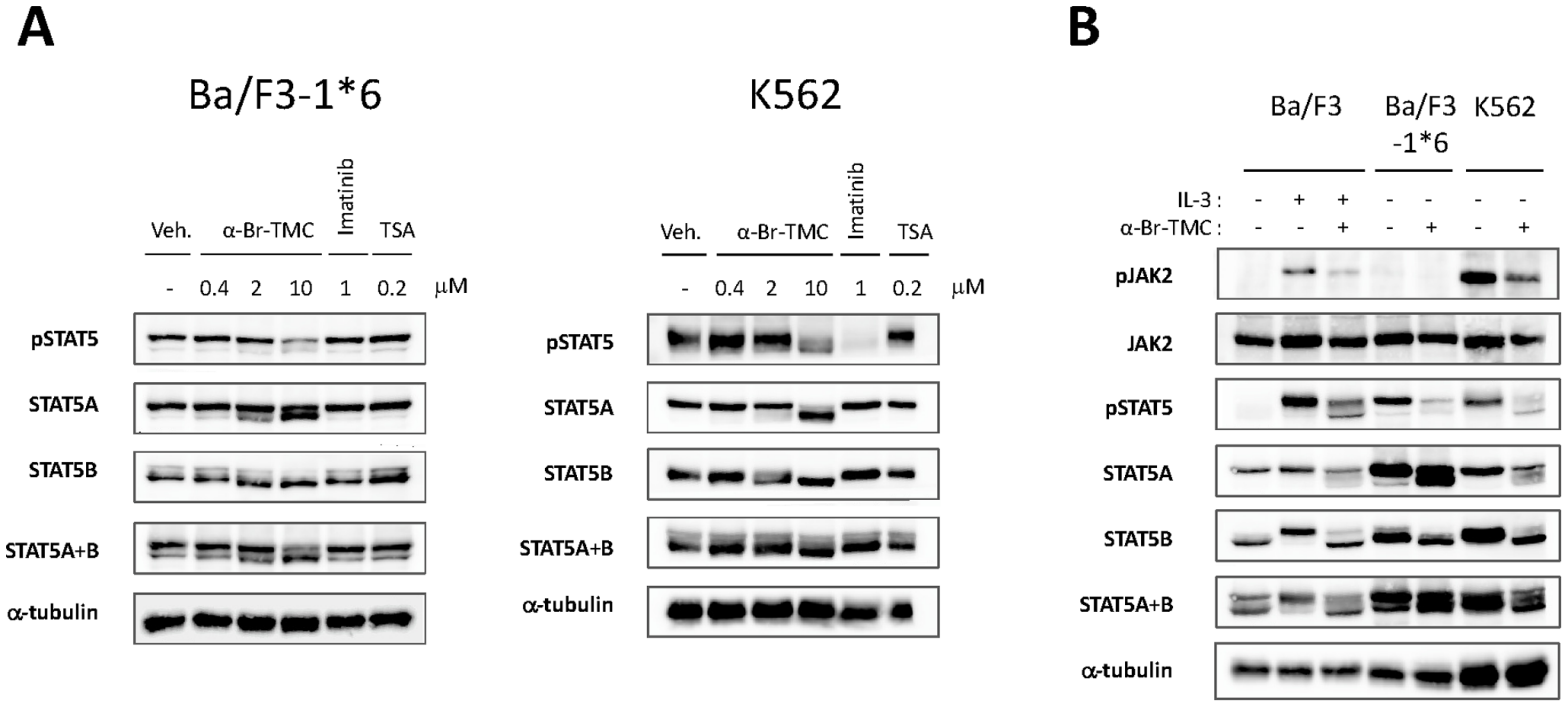
α-Br-TMC inhibits the STAT5 signaling pathway in both a JAK2-dependent and -independent manner. (**A**) Ba/F3-1*6 and K562 cells were treated for 60 minutes with the indicated compounds. Protein whole cell extracts were analyzed by Western-Blot using antibodies specific for pSTAT5, STAT5A, STAT5B, STAT5A and B and α-tubulin as a loading control. (**B**) Ba/F3 cells were pre-treated 30 minutes with 10 µM α-Br-TMC and stimulated with 5 ng/mL IL-3 for 5 minutes. Ba/F3-1*6 and K562 cells were treated with 10 µM α-Br-TMC for 90 minutes. DMSO (Veh.) was adjusted to 0.01% in all conditions. Protein whole cell extracts were analyzed by Western-Blot using antibodies specific for pSTAT5, pJAK2, STAT5A, STAT5B, STAT5A and B, JAK2 and α-tubulin (loading control).

STAT5 phosphorylation in Ba/F3-1*6 and K562 cells was assessed by Western-blot analysis as before, on cells treated with 0.4 to 10 µM α-Br-TMC or 0.2 µM TSA. Both cell lines were also treated with 1 µM Imatinib, as a positive control for pSTAT5 inhibition in K562 cells and a negative control for Ba/F3-1*6 cells (Figure 6A). As expected, TSA did not affect STAT5 phosphorylation in Ba/F3-1*6 and K562 cells. Similarly, the BCR-ABL inhibitor Imatinib did not affect STAT5 phosphorylation in Ba/F3-1*6 cells, while drastically inhibiting STAT5 phosphorylation in K562 cells, as previously reported [75]. As observed in Ba/F3 cells, treatment of Ba/F3-1*6 and K562 cells with α-Br-TMC led to a decrease in STAT5 phosphorylation and a downward mobility shift of both STAT5A and STAT5B proteins, in a dose-dependent manner (Figure 6A and 6B). Again, STAT5B appeared to be more affected than STAT5A.

We next assessed the effect of α-Br-TMC on JAK2 phosphorylation in Ba/F3-1*6 and K562 cells (Figure 6B). In agreement with previous reports [4], [64], pJAK2 was barely detectable in Ba/F3-1*6 cells. The absence of JAK2 phosphorylation in Ba/F3-1*6 cells is also in agreement with our gene expression data, revealing only basal expression levels of the JAK2/MAPK-dependent genes *c-Fos* and *JunB* (Figure 5B). The observation that STAT5 proteins undergo α-Br-TMC-mediated dephosphorylation and mobility shift in the absence of JAK2 supports the idea that these modifications occur in a JAK2-independent manner. Finally, like in Ba/F3 cells, JAK2 phosphorylation in K562 cells was inhibited upon α-Br-TMC treatment (Figure 6B), further indicating that α-Br-TMC targets both JAK2 and STAT5 proteins.

## Discussion

In the search for novel STAT5 inhibitors, we examined the effect of a series of natural and synthetic chalcones as well as of curcumin, another natural α,β-unsaturated carbonyl compound, on JAK2/STAT5 activation and expression of downstream STAT5 target genes in normal (Ba/F3) and caSTAT5-transformed (Ba/F3-1*6, K562) cell lines.

We show here that the synthetic α-Br-2′,3,4,4′-tetramethoxychalcone (α-Br-TMC) is a potent inhibitor of both JAK2 tyrosine kinase and STAT5 transcription factor activities. None of the natural products investigated (curcumin and five natural chalcones) affected STAT5-mediated transactivation of the *Cis* gene in IL-3-stimulated Ba/F3 cells at a concentration of 20 µM. Amongst the four synthetic chalcones α-X-TMCs (with X = CN, Br, Cl, H) tested, α-Br-TMC was the only active STAT5 inhibitor. We showed before that α-Br-TMC, α-Cl-TMC and to a lesser extent α-H-TMC exhibit anti-inflammatory activities in RAW364.7 cells, in particular inhibiting both Nrf2- and NF-κB-dependent pathways [59]. We could not detect an effect of α-Br-TMC on the expression of the Nrf2-dependent *Ho-1* gene in Ba/F3 cells, possibly due to the short duration of treatment. Nevertheless, taken together, our data indicate that α-Br-TMC is able to target multiple pathways controlling inflammation, proliferation and survival. The natural compound sophoraflavanone G was recently reported to exhibit a similar pleiotropic activity against both the JAK/STAT signaling pathway and pathways controlling inflammation and infection in murine and human cell lines [36]. Since interconversion between flavanones and 2′-OH-chalcones was recently evidenced in cell culture [76], it might explain why these two classes of molecules exhibit similar biological activities.

**Figure 7:**
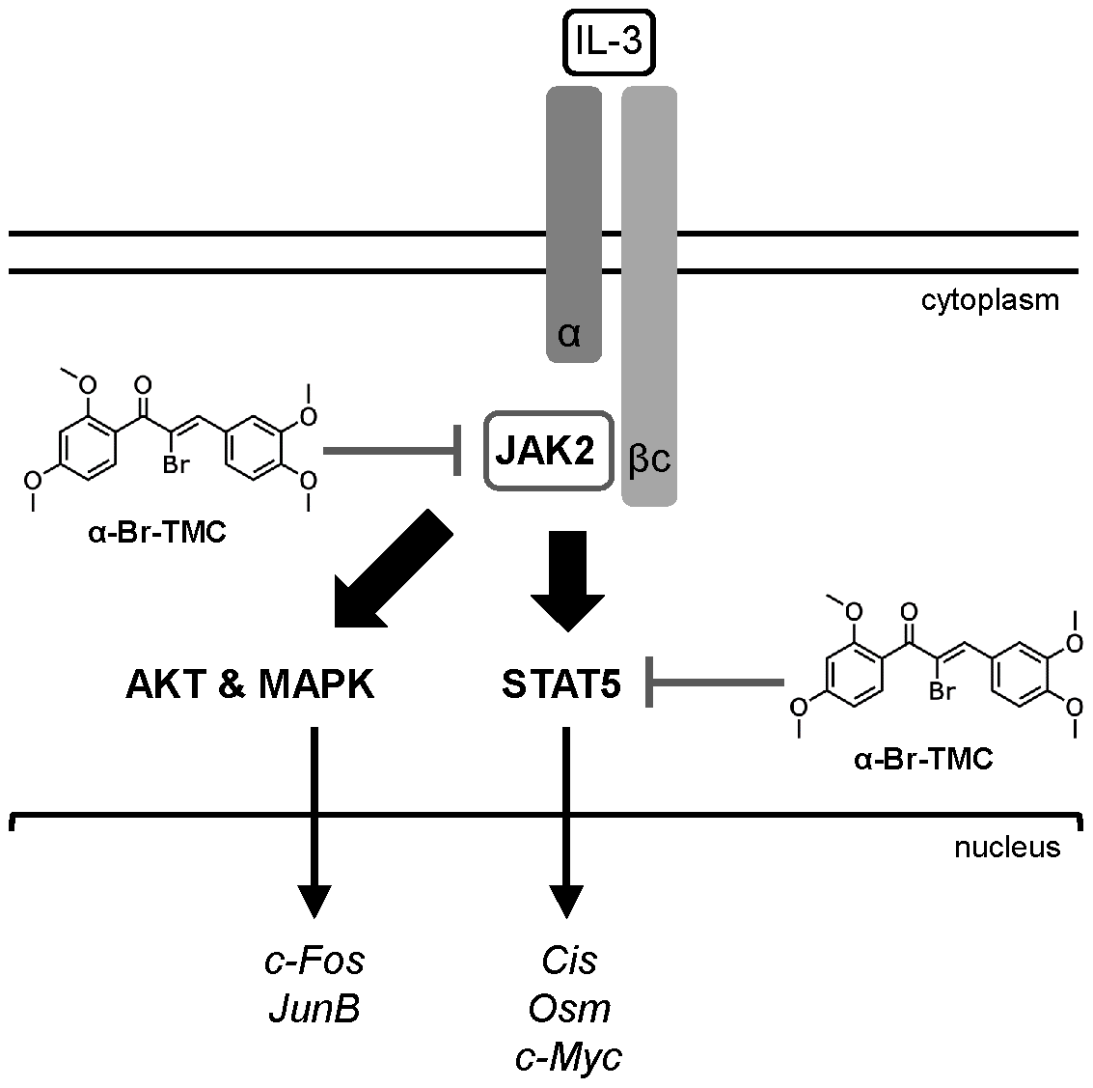
Model of inhibition of the JAK2/STAT5 pathway by α-Br-TMC. IL-3 binding to the α/βc chains of the IL-3 receptor leads to activation of the receptor-associated JAK2 tyrosine kinase by trans-phosphorylation. In turn, JAK2-mediated activation of the STAT5, MAPK and AKT pathways via phosphorylation (broad arrows) results in induced transcription of downstream target genes (thin arrows). We showed that α-Br-TMC inhibits JAK2 phosphorylation, hence impairing JAK2-regulated signaling pathways. In addition and independently of JAK2, α-Br-TMC inhibits STAT5 activity. Concomitantly, STAT5A and STAT5B protein mobility in SDS-PAGE is altered, indicating a change in their post-translational modification state induced by α-Br-TMC, which might be associated to STAT5 altered transcriptional activity.

In accordance with the pleiotropic activity of α-Br-TMC, we found that it targets the JAK2/STAT5 pathway at multiple levels (Figure 7). First, α-Br-TMC inhibits JAK2 tyrosine kinase activation, thus indirectly inhibiting activation of pathways downstream of JAK2, in particular STAT5 and mitogen-activated protein kinase (MAPK). Second, α-Br-TMC inhibits constitutive STAT5 phosphorylation in cells showing basal JAK2 activation (Ba/F3-1*6). Although we cannot rule out that basal JAK2 activation is responsible for the constitutive STAT5 phosphorylation in Ba/F3-1*6 cells [4], our data suggest that α-Br-TMC also inhibits STAT5 phosphorylation - directly or indirectly - in a JAK2-independent manner. Third, α-Br-TMC might alter a post-translational modification on STAT5 proteins, as revealed by a downward mobility shift in SDS-PAGE. α-Br-TMC treatment changed the mobility of both STAT5A and STAT5B proteins in SDS-PAGE, although STAT5B appears more strongly affected than STAT5A. Moreover, this effect takes place within the cytoplasm and independently of IL-3 stimulation and STAT5 phosphorylation. In fact, the faster-migrating STAT5 forms could still be phosphorylated and translocated into the nucleus to some extent. Since this protein modification correlates with a reduced phosphorylation of STAT5A-1*6, possibly independent of JAK2, it is tempting to speculate that α-Br-TMC-induced STAT5 modification interferes - directly or indirectly - with STAT5 activation and hence STAT5 transcriptional activity.

As a result of reduced JAK2 and STAT5 activation, transcription of STAT5 target genes such as *Cis* and *Osm* was impaired, in agreement with a diminished recruitment of RNA polymerase II to their respective transcription start site. Somewhat surprisingly, STAT5 binding to DNA was marginally diminished upon α-Br-TMC treatment. It suggests that the modified nuclear STAT5 proteins still have the ability to bind to DNA and hence might compete with transcriptionally competent unmodified STAT5 proteins.

We found that although STAT5 proteins were similarly modified by α-Br-TMC in normal and transformed cells based on their mobility profile in SDS-PAGE, the consequence on expression of downstream target genes was different. Indeed, while expression of *Cis* and *Osm* was down-regulated in Ba/F3 cells, it was up-regulated in Ba/F3-1*6 and K562 cells. Meanwhile, expression of the *c-Myc* oncogene was reduced in both Ba/F3 and Ba/F3-1*6 cells but slightly increased in K562 cells. According to the differential effect of α-Br-TMC on gene expression, the three cell lines exhibited a distinct chemosensitivity to α-Br-TMC. These observations suggest that α-Br-TMC might target additional signaling and/or transcription factors in cells transformed by constitutively active STAT5 (mouse Ba/F3-1*6 and human K562), resulting in distinct gene expression patterns. The observation that basal expression of *c-Fos* in Ba/F3-1*6 cells in the absence of Jak2 activation was slightly increased upon α-Br-TMC treatment would support this hypothesis. On the other hand, we cannot exclude that α-Br-TMC-mediated modification of STAT5 protein differentially affects STAT5 transcriptional activity in normal and cancer cells. Given that STAT5 DNA binding activity was only weakly impaired in α-Br-TMC-treated cells, one could indeed envisage that DNA-bound STAT5 protein complexes exhibit distinct transcriptional activities depending on the cellular context. For instance, STAT5 is known to regulate transcription through interactions with positive and negative cofactors and with other transcription factors bound on adjacent sites, as well as through tetramerization [1], [9]–[11], which could be differentially regulated in transformed cells. Clearly, additional experiments are needed to determine whether STAT5 is also bound to the *Cis* promoter in Ba/F3-1*6 and K562 cells and further unravel the underlying molecular mechanism.

The question remains as to how α-Br-TMC inhibits JAK2 activation. We cannot rule out at this point that α-Br-TMC acts as a tyrosine kinase inhibitor. Indeed, its structure is reminiscent of that of AG490, a reversible substrate competitive tyrosine kinase inhibitor [77], [78]. In addition, the nature of the protein modification(s) targeting STAT5 upon α-Br-TMC treatment remains to be identified. Several lines of evidences support the idea that the divergent C-terminal transactivation domain of STAT5 is targeted for modification, including the observations that (i) α-Br-TMC inhibited STAT5 phosphorylation at Y694/699, (ii) α-Br-TMC-induced mobility shift of STAT5A was not as pronounced as that of STAT5B, (iii) α-Br-TMC differentially modulated STAT5 transcriptional activity in normal and transformed cells. On the other hand, the observation that STAT5 DNA binding activity was only slightly impaired, raises the possibility that the STAT5 DNA binding domain might be modified as well.

Chalcones in general, and α-Br-TMC in particular, present a highly electrophilic α,β-unsaturated carbonyl moiety that can potentially react with free sulfhydryl groups of cysteine residues in proteins and directly alter their function. On the other hand, electrophiles can inhibit the activation of transcription factors such as NF-κB and STAT3, possibly through S-glutathionylation of cysteine thiol groups, which is itself triggered by a rapid drop of intracellular glutathione [55], [79]. Three naturally occuring terpenes, which also contain an electrophilic α,β-unsaturated carbonyl group, induce S-glutathionylation of STAT3 thereby inhibiting its activity [80], [81]. Similarly to our α-Br-TMC compound, these molecules also inhibit the activation of JAK2 tyrosine kinase [80]. In agreement with the identification of several essential cysteine residues within JAK2 catalytic domain [82], activity of JAK2 - but also of STAT5 - is regulated by the synthetic glutathione disulfide mimetic NOV-002 [83], suggesting that both JAK2 and STAT5 can be S-glutathionylated. Finally, a review of the literature revealed that S-glutathionylation and redox regulation can either stimulate or inhibit protein activity [82], [83], possibly depending on the position of the modified cysteine within the functional domains of the targeted protein. Altogether, these observations raise the possibility that α-Br-TMC, as a potent Michael acceptor, might alter the function of STAT5 and JAK2 proteins, through either direct alkylation or indirect S-glutathionylation of accessible thiol groups, thereby inhibiting or stimulating their activity depending on the cellular context. The presence of cysteine residues in STAT5 proteins, both common and unique to STAT5A and STAT5B within their DNA binding and transactivation domains respectively, is consistent with that model.

On the other hand, the detection of a downward mobility shift of STAT5A and STAT5B upon α-Br-TMC treatment is not in full agreement with the covalent addition of one or several S-glutathione groups. Such downward shift is rather reminiscent of protein dephosphorylation and one could envision an alternative mode of action for α-Br-TMC. Beside tyrosine phosphorylation, STAT5 proteins can be phosphorylated on serine residues [84]. Interestingly, STAT5A and STAT5B are differentially phosphorylated on C-terminal serines (S725/730 in mSTAT5A/B; S779 in mSTAT5A) [84], resulting in either positive or negative transcriptional regulation depending on promoter and/or cellular context [85], [86]. It remains therefore possible that α-Br-TMC either inhibits the still-unknown kinase responsible for serine phosphorylation of STAT5 or activates a specific serine phosphatase, leading to the STAT5 mobility shift detected in SDS-PAGE and possibly to the alteration of its activity.

Whether α-Br-TMC acts as a tyrosine kinase inhibitor and whether the α-Br-TMC-induced STAT5 mobility shift involves S-glutathionylation and/or dephosphorylation of STAT5 and/or of other regulatory components of the STAT5 pathway is currently under investigation.

In conclusion, we identified the synthetic chalcone α-Br-TMC as a novel regulator of JAK2/STAT5 activity, targeting both STAT5 and its upstream activating kinase JAK2 (Figure 7). Its ability to down-regulate expression of the *c-Myc* oncogene while up-regulating expression of the tumor suppressor gene *Cis* in cancer cells, together with its previously described anti-inflammatory properties [59], potentially makes α-Br-TMC a promising novel therapeutic agent with pleiotropic anti-inflammatory and anticancer activities.
