# Correction: The Synthetic α-Bromo-2′,3,4,4′-Tetramethoxychalcone (α-Br-TMC) Inhibits the JAK/STAT Signaling Pathway

**DOI:** 10.1371/journal.pone.0105845

**Published:** 2014-08-08

**Authors:** The PLOS ONE Staff

In Figure 5, some of the graphs are missing numbers on the y-axis. Please see the corrected Figure 5 here.

**Figure 5:**
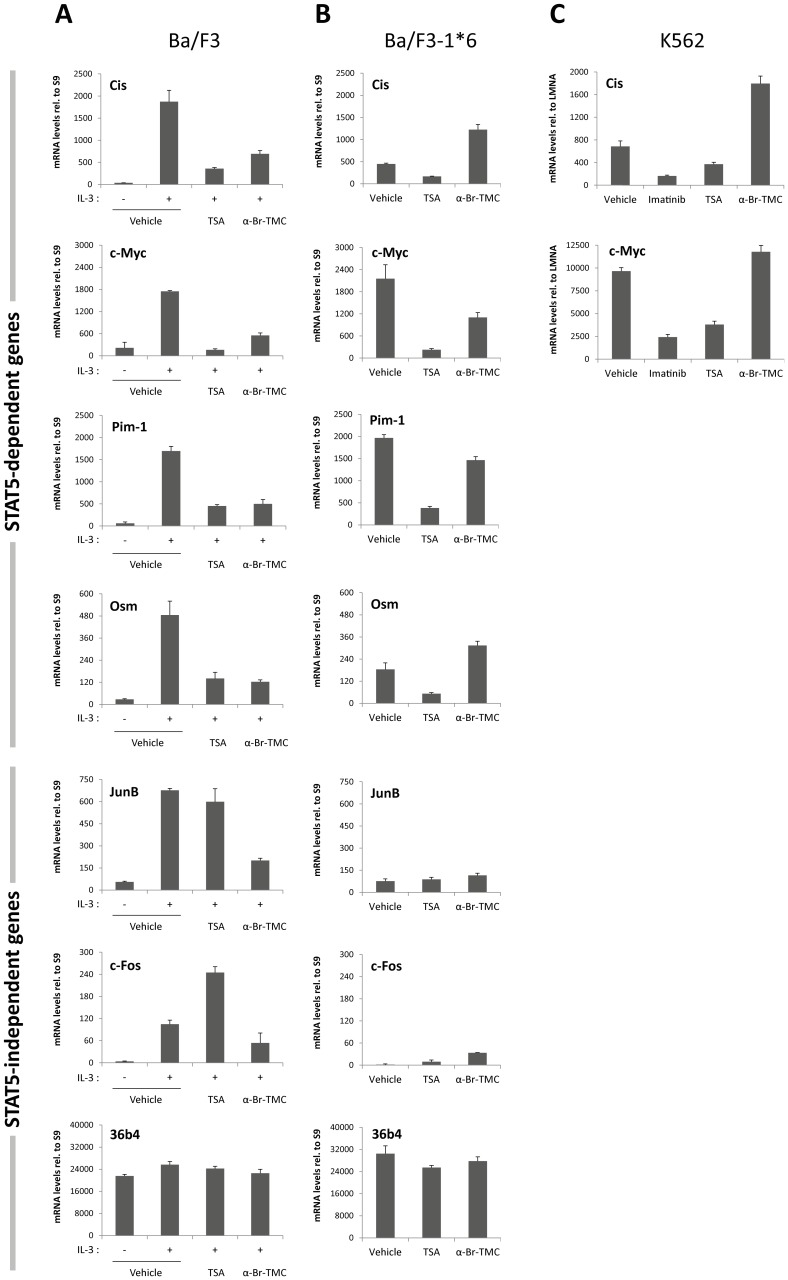
α-Br-TMC exerts distinct effects in normal and cancer cells. Ba/F3 (**A**), its caSTAT5-transformed counterpart Ba/F3-1*6 (**B**) and human leukemic K562 (**C**) cells were treated 90 minutes with 0.2 µM TSA, 10 µM α-Br-TMC or 1 µM Imatinib. Ba/F3 cells (**A**) were stimulated with 5 ng/mL IL-3 after an initial 30 minute drug pre-treatment (hence subjected to a 60 minute IL-3 stimulation). DMSO (vehicle) final concentration was adjusted to 0.02% in all conditions. Expression of STAT5-dependent (*Cis*, *Osm*, *c-Myc, Pim-1*) and -independent (*JunB*, *c-Fos*, *36b4*) genes was analyzed by quantitative RT-PCR. Gene expression data were normalized to mouse ribosomal *S9* (**A, B**) or to human Lamin A/C (*LMNA*) (**C**) housekeeping gene-encoded mRNAs. (**A, B**) Normalized data are presented with adjusted Y-axis scale for a direct comparison of mRNA levels in the respective normal and transformed Ba/F3 and Ba/F3-1*6 cell lines.
